# Study on the change of global ecological distribution of *Nicotiana tabacum* L. based on MaxEnt model

**DOI:** 10.3389/fpls.2024.1371998

**Published:** 2024-07-17

**Authors:** Linxi Jia, Mingming Sun, Mingrui He, Mingfeng Yang, Meng Zhang, Hua Yu

**Affiliations:** ^1^ College of Plant Protection, Shandong Agricultural University, Tai’an, China; ^2^ Technology Center, China Tobacco Shandong Industrial Co., Ltd., Qingdao, China

**Keywords:** *Nicotiana tabacum* L., maximum entropy model, ecological distribution, global warming, habitat change

## Abstract

*Nicotiana tabacum* L. (tobacco) has extremely high economic value, medicinal value, scientific research value and some other uses. Though it has been widely cultivated throughout the world, classification and change of its suitable habitats is not that clear, especially in the context of global warming. In order to achieve rational cultivation and sustainable development of tobacco, current (average from 1970-2000) and future (2070, average from 2061-2080) potential suitable habitats of *Nicotiana tabacum* L. were forecasted with MaxEnt model and ArcGIS platform based on 854 occurrence data and 22 environmental factors in this study. The results revealed that mean temperature of warmest quarter (bio10), annual precipitation (bio12), solar radiation in September (Srad9), and clay content (CLAY) were the four decisive environment variables for the distribution of *Nicotiana tabacum* L. Under current climate conditions, suitable habitats of *Nicotiana tabacum* L. were mainly distributed in south-central Europe, south-central North America, most parts of South America, central Africa, south and southeast Asia, and southeast coast of Australia, and only 13.7% of these areas were highly suitable. By the year 2070, suitable habitats under SSP1-2.6, SSP3-7.0, and SSP5-8.5 climate scenarios would all increase with the largest increase found under SSP3-7.0 scenario, while suitable habitats would reduce under SSP2-4.5 climate scenario. Globally, the center of mass of suitable habitats would migrate to southeast to varying degrees within Libya under four different climate scenarios. The emergence of new habitats and the disappearance of old habitats would all occur simultaneously under each climate scenario, and the specific changes in each area, combined with the prediction results under current climate conditions, will provide an important reference for the adjustment of agronomic practices and rational cultivation of *Nicotiana tabacum* L. both currently and in the future.

## Introduction

*Nicotiana tabacum* L. (tobacco), a genus of plants in the Solanaceae family, is the primary raw material used in the cigarette production. It also serves as an indicator plant to test for sulfur dioxide and nitrogen oxide pollution. *Nicotiana tabacum* L. can also serve as a model crop for scientific researches because of its fast growth rate, simplicity of laboratory culture, and its large cells which are easy to genetically engineer (Zou et al., 2021). Many extracts from *Nicotiana tabacum* L. also have great medical value (Liu et al., 2012; Sari and Khalil, 2015; Yao et al., 2015). *Nicotiana tabacum* L. plays an important role in many fields.

Environment conditions have a significant impact on the quality and yield of tobacco. Though *Nicotiana tabacum* L. has been widely cultivated worldwide within a latitudinal and longitudinal distribution range from 55°N to 40°S, its most suitable planting zone is very limited (Pollastri et al., 2023). Brazil, Zimbabwe, Zambia, America, and India are the main producing areas of high-quality tobacco (Martins-Da-Silva et al., 2022). These tobacco leaves are widely favored by the cigarette industry, and these countries therefore become the main tobacco exporting countries obtaining huge economic profits. To further identify classification of suitable habitats and then look for new high-quality tobacco producing areas and cope with the impact of global warming as well, the distribution of tobacco in different time ranges and under different climatic conditions deserves a comprehensive study.

One classical way to assess the ecological adaptation of species is to use a species distribution model (SDM) to estimate the likely habitats of species based on limited distribution samples (Guo et al., 2015; Waldock et al., 2022). Many SDMs have been widely used, such as maximum entropy theory (MaxEnt), Random Forest (RF), Boosted Regression Tree (BRT), Bioclim, Generalized Linear Model (GLM) and CLIMEX (CL) (Booth et al., 2014; Duan et al., 2014; Shabani et al., 2016). Among these SDMs, MaxEnt, proposed based on the maximum entropy theory, offers superior accuracy and reproducibility, at the same time, it is easier to operate and does not require expensive computing resources (Ahmadi et al., 2023; Norberto et al., 2023). Hence, MaxEnt is highly regarded by researchers and many habitat predictions of plants and animals are made using it in recent years, such as Agastache rugosa, soybean, Hylomecon japonica, Buckwheat, Scutellaria baicalensis, and Rainfed Maize, etc (Pearson et al., 2006; Kogo et al., 2019; Xu et al., 2020; Wen et al., 2021; Cuddington et al., 2022; Gong et al., 2022; Wang et al., 2023). Additionally, MaxEnt has also been used in the study of *Nicotiana* L. For example, our team previously uses MaxEnt to predict the appropriate habitats for *Nicotiana alata* Link et Otto worldwide, and Maguranyanga C concludes that MaxEnt can be used to map tobacco fields in Zimbabwe using a time series of Normalized Difference Vegetation Index (NDVI) data in ecological suitability analysis (Maguranyanga and Murwira, 2014; Zhang et al., 2023). Therefore, it is also feasible to utilize MaxEnt to investigate the global ecological distribution of *Nicotiana tabacum* L. here.

In this study, in order to clarify the classification and change of suitable habitats to provide theoretical support for rational cultivation and sustainable development of *Nicotiana tabacum* L., especially in the context of global warming, current (average from 1970-2000) and future (2070, average from 2061-2080) potential suitable habitats of *Nicotiana tabacum* L. were forecasted with MaxEnt model and ArcGIS platform based on large amounts of occurrence data and multiple corresponding environmental factors. The results of regional prediction and analysis of key environmental factors will provide important references for the discovery of high-quality tobacco planting areas and the rational cultivation of tobacco, both now and in the future.

## Materials and methods

### Collection and rasterization of the occurrence records of *Nicotiana tabacum* L.

Global occurrence records of *Nicotiana tabacum* L. came from the Global Biodiversity Information Facility (GBIF, https://www.gbif.org/), literature records and field survey (Ghosh et al., 2014; Grisan et al., 2016; Song et al., 2016; Ruiz-Vera et al., 2017; Yang et al., 2018; Wang et al., 2020; Zheng et al., 2020; Hu et al., 2021; Sifola et al., 2021; Kulesza et al., 2022; Brizuela-Fuentes et al., 2023; GBIF.org, 2023; Huang et al., 2023). A total of 3684 samples were obtained at first (3650 samples from GBIF, 18 samples from literatures, and 16 samples from our field survey in Shandong Province of China). Then, because these occurrence records come from sample records of collections from different regions, literatures and field survey, some of the collection data have a high degree of similarity on the maps, which can lead to overfitting of the model and affect the model results (Gao et al., 2023). Thus, the spThin package’s thin function was employed in the R studio software to thin the data using the distance threshold method, leaving 854 sample records after screening (Low et al., 2021; Zhang et al., 2021).

### Screening of environmental variables

A total of 56 environment variables related to tobacco growth were collected, including 19 bioclimatic variables, 22 soil factors, 3 topographic factors and 12 solar radiation factors. Data of nineteen bioclimatic variables, Digital Elevation Model (DEM) and twelve solar radiation factors were obtained from the latest climate variable layers provided by the WorldClim Global Climate Database (version 2.1) (Fick and Hijmans, 2017) (http://www.worldclim.org/). DEM is one of the three topographic factors, and the other two factors are Slope and Aspect which were obtained after processing DEM data with Arcgis10.8. Data of 22 soil factors were obtained from Harmonized World Soil Database v2.0 (https://www.fao.org). The grid resolution for all environment variable layers was 2.5 min.

The environmental data of 854 sample records with 56 strata were extracted and further transported to the Pearson correlation test in SPSS for correlation screening, where variables with high correlation (P > 0.7) were eliminated and 22 variables with the lowest dependency were finally obtained for the calculation of the MaxEnt model (**Supplementary Table S1**) (Sharma et al., 2018; Sutton, 2019).

### Future climate conditions

BCC-CSM2-MR, one of three models of the Beijing Climate Center Climate System Model in the Coupled Model Intercomparison Project Phase 6 (CMIP6), was used with four Shared Socio-economic Pathways (SSPs) for the year 2070 (average from 2061-2080) which named SSP1-2.6, SSP2-4.5, SSP3-7.0, and SSP5-8.5 in this study (Pincus et al., 2016; Beijing Climate Center, 2019; Meinshausen et al., 2020). The data were obtained from the open access website (https://worldclim.org/). The SSP1-2.6 scenario denotes a sustainable development path with a warming of approximately 2°C, whilst the SSP2-4.5 scenario denotes a medium development path with about 3°C warming. The SSP3-7.0 scenario represents a local development path that restricts the warming to about 4.2°C, and the SSP5-8.5 scenario involves a traditional development path that restricts the temperature increase to about 5°C (Parmesan and Yohe, 2003; Lenoir et al., 2008).

### MaxEnt model and ArcGIS platform

Maxent software (Version 3.4.1) used in this study was obtained from: https://biodiversityinformatics.amnh.org/open_source/maxent/ (Phillips et al., 2017).

Based on the need to process species data and environmental variables, a comprehensive GIS system, ArcGIS 10.8, was used. ArcGIS for Desktop is a complete set of professional GIS applications (Kalboussi and Achour, 2018), of which ArcMap, ArcCatalog, and ArcTool box are the core components. ArcMap can be used for sampling, reclassification, and raster data import, and is the main application used for mapping, editing, analysis, and data management (Yu et al., 2019).

### Model building of MaxEnt

**Figure 1:**
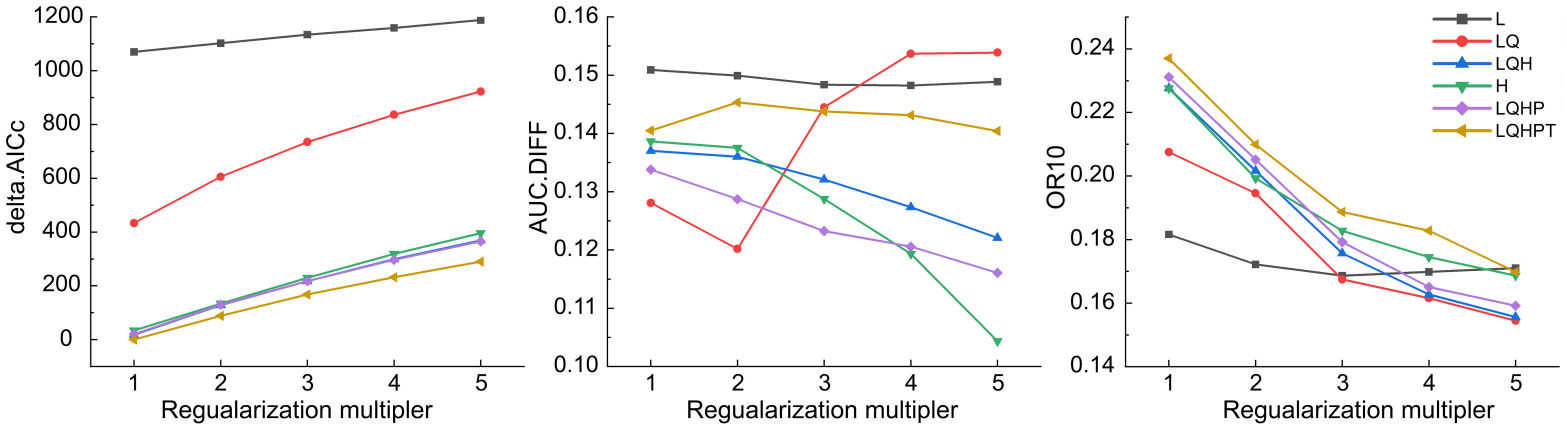
MaxEnt assessment indicators generated by ENMeval.

The calculation of the global ecological distribution of *Nicotiana tabacum* L. was carried out using MaxEnt. The 854 sample records and their environmental layers which have been converted into ASCII format using ArcGIS 10.8 software were imported into MaxEnt 3.4.1 software for calculation. Linear (L), quadratic (Q), hinge (H), product (P), and threshold (T) are the five parameters of MaxEnt software (Morales et al., 2017). The accuracy of the model will change with different parameters for different species (Radosavljevic and Anderson, 2014; Li et al., 2020). To simplify the model and improve reliability, we used the ENMeval software to optimize parameter settings. Akaike information criterion correction (AICc), the difference between training and test AUC (AUC.diff) and 10% training omission rate (OR10) were utilized to evaluate the model’s fitting degree (Yu and Yau, 2012). The results showed that when the regularization multipliers (RM) equaled to 1 and that the feature combination (FC) was the combination of LQHPT, the delta.AICc was 0, and the fitting degree of the model was high (**Figure 1**) (Elith et al., 2010). Then, open the MaxEnt model software and set parameters in Setting. 75% and 25% were the data range criteria of the training set and the test set respectively (Sillero et al., 2023). The maximum number of background points was 10000. The replicates were 10. Then, click “Run” to start the data simulation. The output file format was “asc”, the type was Logistic, and other parameters were default. Finally, a total of 777 sample records were used for calculation (**Supplementary Figure S1**), as MaxEnt automatically deleted some sample records with incomplete data on environmental variables.

### Model reliability and accuracy

AUC (Area Under Curve) is defined as the region situated beneath the ROC (receiver operating characteristic) curve enclosed by the axis. This value indicates the accuracy of the predictive model, with a higher value closer to 1 representing a better prediction accuracy (Duan et al., 2022; Zhao et al., 2023). An AUC value of 0.85-0.95 can be considered a very good prediction.

The True Skill Statistic (TSS) was also used to evaluate the accuracy of the MaxEnt model. The value of TSS ranges from -1 to 1, with a value of 0.6 to 1 indicating good results, and a value closer to 1 indicating higher accuracy (Swets, 1988).

Another evaluation indicator is the continuous Boyce index (CBI) (Lake et al., 2020). The value of CBI is from -1 to 1. Positive values of CBI demonstrate that the distribution predicted by the model agrees with the observed data, and the closer it is to 1, the more reasonable the prediction is. A result close to 0 suggests a stochastic prediction model, while a negative outcome indicates unsatisfactory prediction results (De Gasper et al., 2021).

### Classification of suitable habitats of *Nicotiana tabacum* L.

The simulation results were visualized and classified into different levels of suitable habitats using ArcGIS 10.8 software: select the avg layer in the model result file, import the raster data into the software, perform the classification using the Jenks method and then obtain four classes: unsuitable class (<0.09), lowly suitable class (0.09-0.26), moderately suitable class (0.26-0.47), and highly suitable class (>0.47). Open the attributes panel of the layer, use the built-in field analysis tool of ArcGIS 10.8 to count and calculate the area of the corresponding distribution region for each habitat layer (Zhou et al., 2021; Aouinti et al., 2022).

## Results

### Model reliability and accuracy

**Figure 2:**
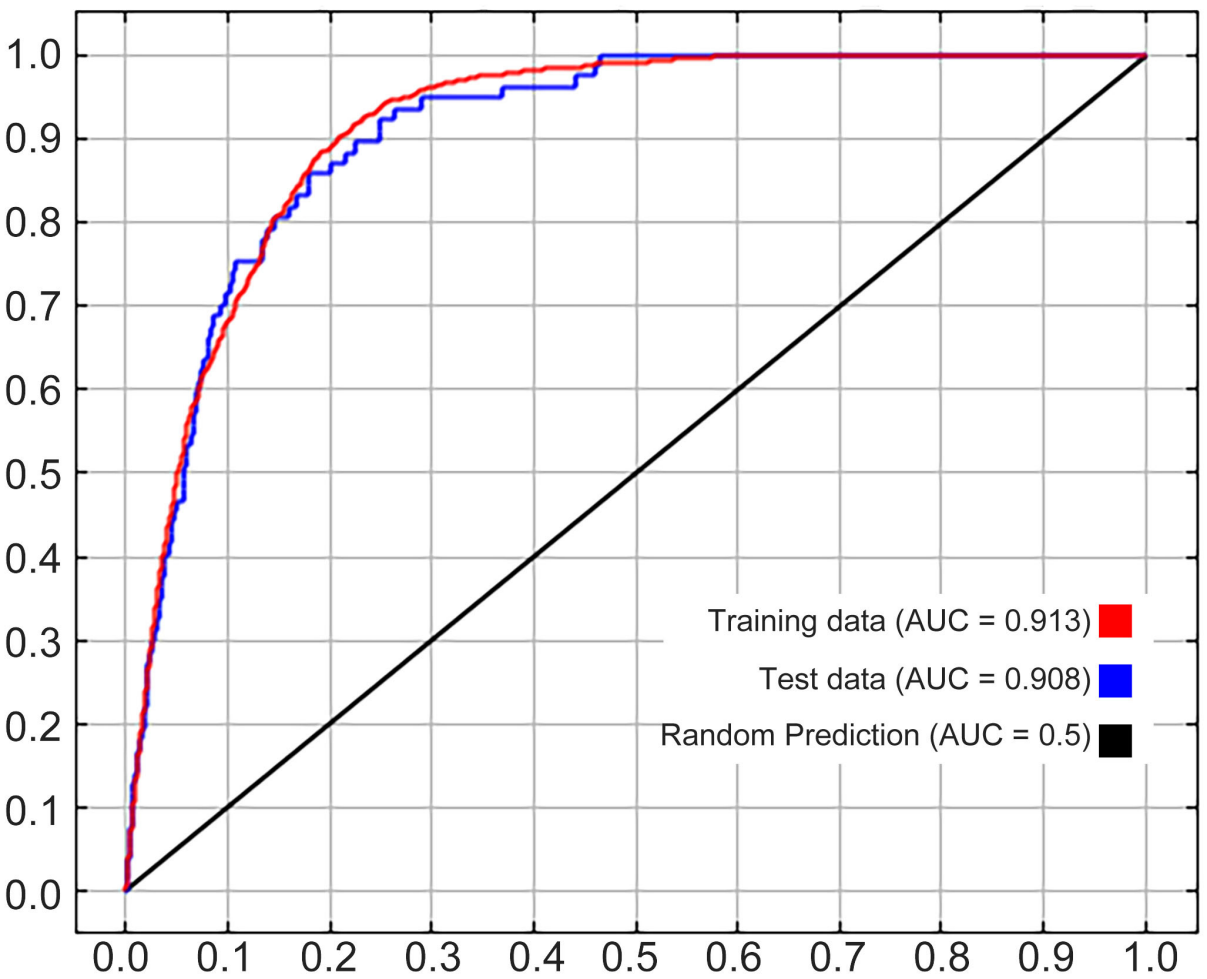
AUC values under the ROC curve.

AUC, TSS, and CBI were calculated to measure the reliability and accuracy of our MaxEnt. The calculations illustrated that (**Figure 2**) the AUC values of the test set and the training set were 0.908 (**Figure 2**, area under the blue line) and 0.913 (**Figure 2**, area under the red line) respectively, and the CBI result was 0.852. After ten runs of the MaxEnt model, the calculated average TSS was 0.726. Both AUC values and the CBI value were not less than 0.85 and the average TSS was in the range of 0.6 to 1, suggesting that our MaxEnt model was rather reliable and its accuracy in calculating the ecological distribution of *Nicotiana tabacum* L. on a global scale would be very high.

### Influence of environment variables on the construction of the MaxEnt model

The influence of each environment variable on the construction of the model was explored. The results (**Supplementary Table S2**) showed that mean temperature of warmest quarter (bio10), annual precipitation (bio12), and solar radiation in September (Srad9) were the top three key environment variables both from the perspective of percentage contribution and permutation importance. Besides, the cumulative percentage contribution and permutation importance of these three key variables were 65.6% and 64.9% respectively, both of which were more than half, further suggesting their importance for the construction of the model. In addition, the cumulative percentage contribution of bioclimatic variables, soil variables, topographic variables and solar radiation variables were 70.9%, 17.4%, 3.7%, and 7.9% respectively, demonstrating the significant importance of bioclimatic variables. And at the same time, the cumulative permutation importance of bioclimatic variables, soil variables, topographic variables and solar radiation variables were 63.5%, 6.3%, 16.2%, and 14.2% respectively, which also indicated the most importance of bioclimatic variables. Taken together, the results suggested that bioclimatic variables were much more important than soil, topographic, and solar radiation variables and that bio10 (mean temperature of warmest quarter), bio12 (annual precipitation) and Srad9 (solar radiation in September) were the three key individual environment variables that have a great impact on the construction of the MaxEnt prediction model.

### Impact of environment variables on the distribution of *Nicotiana tabacum* L.

**Figure 3:**
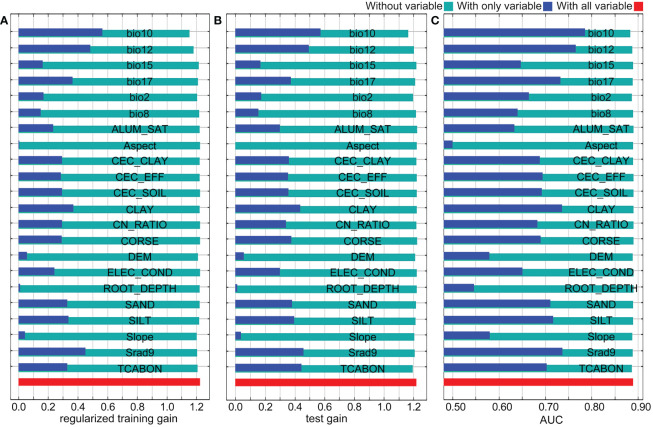
Jackknife test of the MaxEnt model. **(A)** The regularized training gain. **(B)** The test gain. **(C)** The AUC.

The impact of environment variables on the distribution of *Nicotiana tabacum* L. was analyzed using the jackknife method (**Figure 3**). In the knife-cut results, the blue bar indicates only this variable, and the higher the training score, the bigger the effect this variable makes on the abundance of species. The green bar represents that this variable is not included, and a lower training score indicates that this variable contains more specific information. In the regularized training gain, bio10, bio12, and Srad9 were the three variables that had the greatest influence on the distribution of *Nicotiana tabacum* L. with gain values all greater than 0.4, while CLAY and bio17 were the two variables with great influence on the distribution and their gain values were slightly less than 0.4 (**Figure 3A**, blue bars). Bio10 and bio12 were the two variables containing the most specific information with gain values less than 1.2 (**Figure 3A**, green bars). In the test gain, bio10, bio12, Srad9, TCABON, and CLAY were the five variables that had the greatest influence on the distribution of *Nicotiana tabacum* L. with gain values all greater than 0.4 (**Figure 3B**, blue bars). Bio10 was the variable containing the most specific information with a gain value less than 1.2, while TCABON, bio2, and bio12 were the three variables containing more specific information with gain values very close to 1.2 (**Figure 3B**, green bars). In the AUC results, bio10, bio12, Srad9, and CLAY had the top four AUC values when testing with only this variable (**Figure 3C**, blue bars), indicating their importance for the distribution of *Nicotiana tabacum* L. Meanwhile when testing without this variable, only bio10, TCABON, bio2, bio12, and Slope slightly reduced AUC values and AUC values shown with green bars were all greater than 0.85, both of which verified the reliability of the model (**Figure 3C**, green bars). Overall, the results of the knife-cut analysis showed that bio10 (mean temperature of warmest quarter), bio12 (annual precipitation), Srad9 (solar radiation in September), and CLAY (clay content) were the key environmental variables influencing the distribution of *Nicotiana tabacum* L.

**Figure 4:**
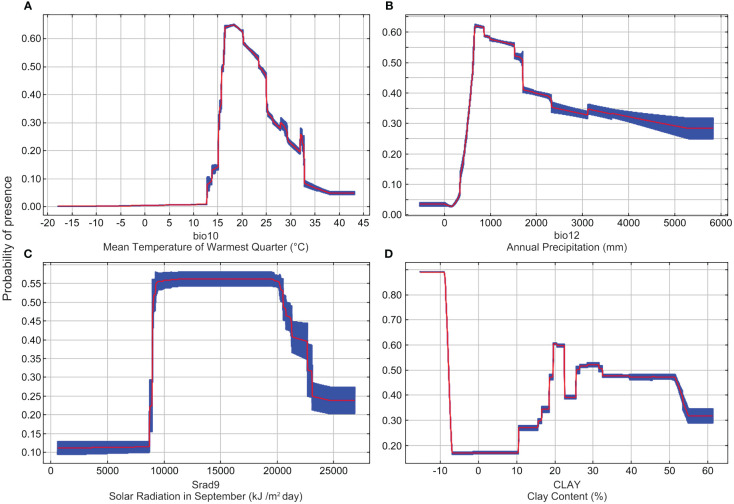
Response curves of four decisive environmental variables. **(A)** The response curve of mean temperature of warmest quarter (bio10). **(B)** The response curve of annual precipitation (bio12). **(C)** The response curve of solar radiation in September (Srad9). **(D)** The response curve of clay content (CLAY).

In order to better grow *Nicotiana tabacum* L., further investigations were carried out to obtain the highly suitable values of the above four key environmental variables (bio10, bio12, Srad9, and CLAY). A presence probability greater than 0.47, the same range with highly suitable habitats, was used to estimate the range of the highly suitable value for each variable. The results showed that the highly suitable value range of bio10 was 16-25°C (**Figure 4A**). The presence probability of *Nicotiana tabacum* L. gradually increased in the range of 13-17°C, and then gradually decreased in the range of 17-30°C (**Figure 4A**). The highly suitable value range of bio12 was 600-1700 mm (**Figure 4B**). The presence probability increased from 200-800 mm and then decreased after bio12 reached 800 mm (**Figure 4B**). The highly suitable value of Srad9 was between 9000 kJ/m^2^day and 21000 kJ/m^2^day (**Figure 4C**), and the highly suitable value of CLAY was between 19% and 23% and between 26% and 52% (**Figure 4D**).

### Global ecological distribution under present environmental conditions

**Figure 5:**
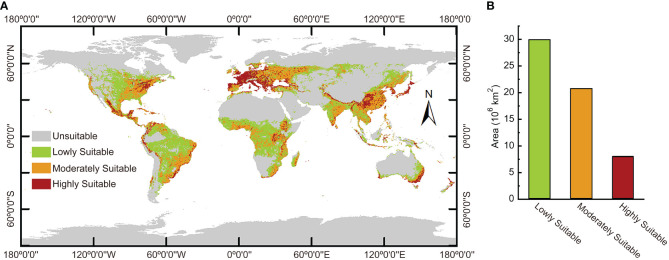
The global ecological distribution of *Nicotiana tabacum* L. under present environmental conditions. **(A)** Geographical distribution of different ecological regions. **(B)** The area of different ecological regions.

The MaxEnt model was used to calculate the ecological range of *Nicotiana tabacum* L. globally under present climate circumstances. The calculation results (**Figure 5A**) showed that the most suitable areas to plant *Nicotiana tabacum* L. were mainly in the most parts of southern and western Europe, eastern United States, Mexico, coastal areas of northwest and southeast South America, central and eastern Africa, southwest China, the Korean Peninsula, Japan, the southeast coast of Australia, and so on. The moderately suitable areas were concentrated in east-central United States, southeast South America, central Europe, west Africa, eastern India, and southeast and northeast China. The moderately suitable areas were generally distributed around the highly suitable areas and spread outward. The lowly suitable areas were large and mainly located in central United States, central and northern South America, central Africa, central Russia, northeast China, western and northern India, southeast Asia, and southeast Australia. In general, the highly suitable areas were distributed in spots, bands, and clumps, while the moderately and lowly suitable areas were distributed in sheets and blocks. The highly suitable habitats covered 8.0778×10^6^ km^2^, accounting for 13.7% of the total habitats. The moderately suitable habitats spanned 2.07719×10^7^ km^2^, accounting for 35.3% of the total habitats. And the lowly suitable habitats covered 2.99446×10^7^ km^2^, accounting for 50.9% of the total habitats (**Figure 5B**).

### Change of global ecological distribution under four different climate scenarios in the future

**Figure 6:**
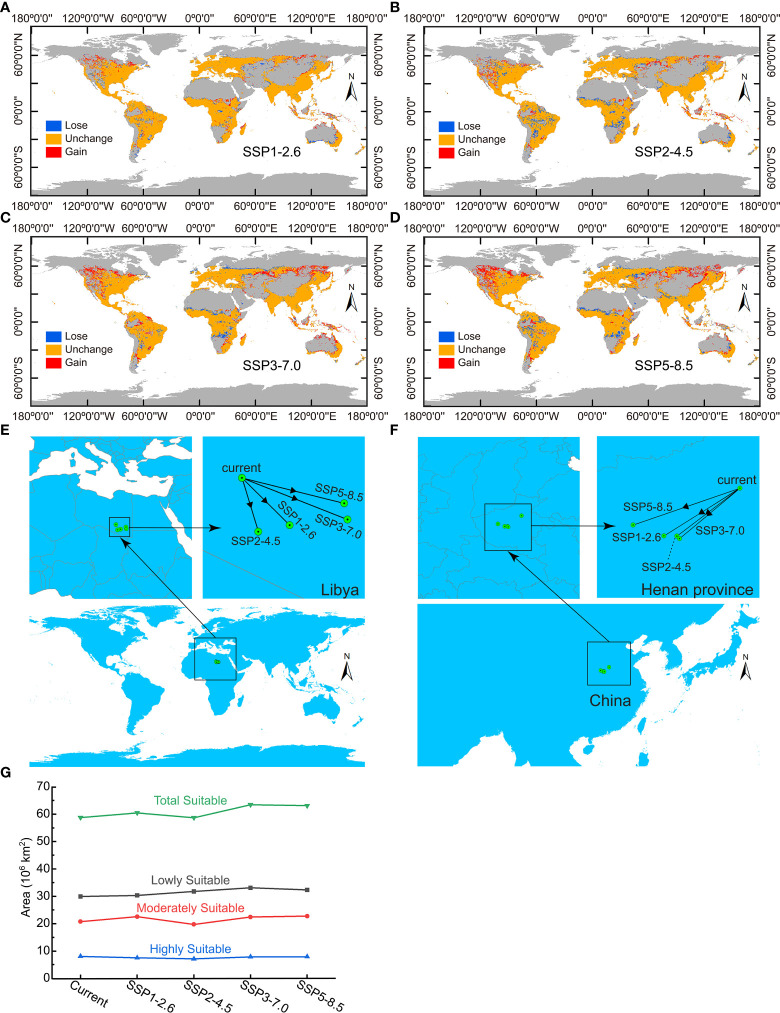
Change of global ecological distribution of *Nicotiana tabacum* L. under four future climate conditions. **(A)** Ecological distribution change under SSP1-2.6 climate scenario. **(B)** Ecological distribution change under SSP2-4.5 climate scenario. **(C)** Ecological distribution change under SSP3-7.0 climate scenario. **(D)** Ecological distribution change under SSP5-8.5 climate scenario. **(E)** Migration of the center of mass of the world’s habitat. **(F)** Migration of the center of mass of the habitat within China. **(G)** Comparison of the area of different ecological regions among all of climate conditions.

Global ecological distribution of *Nicotiana tabacum* L. under four future climate conditions were also calculated, and then, distribution changes between present and future climate conditions were obtained to clearly show the change of global ecological distribution in the future (**Figure 6**). The changes indicated that the future distribution areas of *Nicotiana tabacum* L. indeed had some difference from the situation under present climate conditions. Geographically, the newly added suitable areas (gain in **Figure 6**) were mainly concentrated in central North America and high latitudes in Asia with large area gains under SSP3-7.0 and SSP5-8.5 climate scenarios and small gains under SSP1-2.6 and SSP2-4.5 climate scenarios. At the same time, there were new lost areas, mainly located on the border of Europe and Asia, Africa and South America. The lost area was also large under SSP3-7.0 and SSP5-8.5 climate scenarios and relatively small under SSP1-2.6 and SSP2-4.5 climate scenarios. On the whole, compared to the area under current climate conditions, the total global potential suitable area of *Nicotiana tabacum* L. in 2070 (average from 2061-2080) increased by 1.7043×10^6^ km^2^, 4.6952×10^6^ km^2^, 6.3135×10^6^ km^2^ under SSP1-2.6, SSP3-7.0 and SSP5-8.5 scenarios respectively, and decreased by 6.6424×10^5^ km^2^ under SSP2-4.5 scenario (**Figure 6G**). Using the center of mass (CoM) of the global habitat under current climate conditions as a reference (20.652033°E, 23.508065°N), the CoM under four future climate scenarios were all migrated to the southeast within Libya with the migration distance of 189.08 km, 163.62 km, 309.67 km, and 285.02 km under SSP1-2.6, SSP2-4.5, SSP3-7.0, and SSP5-8.5 climate scenarios, respectively (**Figure 6E**). The CoM eventually migrated to 21.910274°E and 22.263944°N, 21.083727°E and 22.091412°N, 23.431285°E and 22.409326°N, and 23.342818°E and 22.836192°N under SSP1-2.6, SSP2-4.5, SSP3-7.0, and SSP5-8.5 climate scenarios, respectively (**Figure 6E**).

Within China, habitat gains occurred in the north and northwest regions, while habitat loss was also concentrated in the above regions, except for very slight habitat loss in the southwest, east, and southeast regions. Under SSP1-2.6 climate scenario, habitat gains occurred mainly in Inner Mongolia province and some habitat loss occurred in northeastern Inner Mongolia, Heilongjiang, and Jilin provinces (**Supplementary Figure S2A**). Under SSP2-4.5 climate scenario, the most prominent phenomenon was the loss of the habitat which was mainly in Inner Mongolia, Heilongjiang, and Jilin provinces (**Supplementary Figure S2B**). Under SSP3-7.0 and SSP5-8.5 climate scenarios, habitat gains mainly occurred in Inner Mongolia, Ningxia, and Gansu provinces, and the gain was much more under SSP5-8.5 climate scenario, suggesting the possibility of expanding planting in large area (**Supplementary Figures S2C**, **D**). Compared with the habitat loss under SSP1-2.6 and SSP2-4.5 scenarios, small scale habitat loss also occurred in Sichuan, Yunnan, and Taiwan provinces under SSP3-7.0 climate scenario (**Supplementary Figure S2C**), and further habitat loss occurred in Shandong, Jiangsu, and Guangxi provinces under SSP5-8.5 climate scenario (**Supplementary Figure S2D**). Though the loss of the habitat in the southwest, east, and southeast regions of China seemed very small, it was a depressing result for China’s tobacco industry as these regions were highly or moderately suitable areas for tobacco (**Figure 5A**; **Supplementary Figure S3**) and do in fact produce high-quality tobacco especially for Yunnan and Sichuan. Using the CoM of the habitat in China under current climate conditions as a reference (114.108627°E, 34.722362°N), the CoM under four future climate scenarios were all migrated to the southwest within Henan province with the migration distance of 146.80 km, 131.69 km, 132.09 km, and 177.53 km under SSP1-2.6, SSP2-4.5, SSP3-7.0, and SSP5-8.5 climate scenarios, respectively (**Figure 6F**). The CoM eventually migrated to 112.838821°E and 33.920372°N, 113.055564°E and 33.918438°N, 113.099849°E and 33.875793°N, and 112.324604°E and 34.103538°N under SSP1-2.6, SSP2-4.5, SSP3-7.0, and SSP5-8.5 climate scenarios, respectively, with a trend of migration to lower latitudes (**Figure 6F**).

## Discussion

*Nicotiana tabacum* L. is a very important plant with huge economic, medicinal and chemical value. This study revealed suitable areas for the growth of *Nicotiana tabacum* L. worldwide under current climate conditions and then changes of suitable areas in the future. Several important environment variables controlling the growth and distribution of *Nicotiana tabacum* L. were also investigated in detail. The results of this study provide effective guidance for the rational production of *Nicotiana tabacum* L. at present and in the future.

Environmental factors are crucial in the growth of species. It is reported that temperature and precipitation are the most critical ones among all of the environmental factors (Liu et al., 2023). Variations in temperature and precipitation can affect plant physiological processes, including nutrient growth and reproductive growth at different latitudes and longitudes. Besides, temperature changes can affect soil water content, nutrient uptake and utilization (Ding et al., 2020), therefore, temperature can have a direct impact on the growth and distribution of plants by influencing their morphology, physiology, chemistry, and biochemical activities. Consistently, in our results, bioclimatic variables were generally much more important than soil, topographic and solar radiation variables for the distribution of *Nicotiana tabacum* L. (**Supplementary Table S2**). Detailed analysis showed that temperature, precipitation, solar radiation and clay content related (bio10, bio12, Srad9, CLAY) environment variables were the four key factors affecting distribution, and finally the temperature and precipitation related bioclimatic variables bio10 and bio12 were the most critical ones (**Figure 3C**). Further, it can be imagined that changes in mean temperature of warmest quarter (bio10) and annual precipitation (bio12), which will change significantly with the global warming, will also be the cause of habitat changes in the future (**Figure 6**). Last but not least, the result of this study also agrees with the growing habits of *Nicotiana tabacum* L. that it prefers warm, sunny environments, and that it is not cold tolerant, more heat tolerant (Liang et al., 2022).

Each specific bioclimatic variable affects different species in different ways, even within the same genus, which may be due to differences in their own growth habits. For example, in the same genus of *Hordeum* L., in Weidong Ma’s study, the bioclimatic variables bio6, bio12, bio3 and bio9 are important bioclimatic variables influencing the distribution of highland barley (*Hordeum vulgare* var. *trifurcatum*), whereas in Paul Evangelista’s study, the distribution of barely (*Hordeum vulgare* var. *coeleste* Linnaeus) was found to be influenced by bioclimatic variables, specifically the bioclimatic variables bio12 and bio16 (Evangelista et al., 2013; Yin et al., 2022). In the same genus of *Nicotiana* L., our previous study on *Nicotiana alata* Link et Otto showed that the bioclimatic variables bio9, bio14, bio15, and bio5 are the decisive factors for suitable habitats (Zhang et al., 2023), whereas, the study on *Nicotiana tabacum* L. in this paper revealed a completely different result that the environment variables bio10, bio12, Srad9 and CLAY (**Figure 3C**) are the decisive ones. Again, these study results suggest that each particular species needs to be studied specifically, and results from any other species cannot be used as a reference.

Prediction for *Nicotiana tabacum* L. under current climate conditions showed that Western and Southern Europe, in the northern temperate zone, has the highest concentration of highly suitable habitats (**Figure 5A**). This is closely related to their unique climate. Western Europe is located on the eastern coast of the Atlantic Ocean and experiences a temperate oceanic climate. In contrast, Southern Europe enjoys a Mediterranean climate, but Eastern and Central Europe, as well as much of the rest of the continent, are situated far inland and have a temperate continental climate. In regions with oceanic climates, the closer you are to the oceans, the more oceanic the climate is. In winter, the air currents become warmer and more humid due to the passage of warm currents along the coast and westerly winds blowing from the warmer seas. As a result, winter temperatures in western and southern Europe are above 0 degrees, much higher than the center and eastern part of the continent at the same latitude. In summer, the sea is much cooler than the land, although the water temperatures of the warm currents are still cooler than the continental temperatures. Here, the average temperature in the hottest months is below 22°C due to the westerly winds, the annual precipitation is more than 800 mm. Because of the warm winters and cool summers, the annual temperature difference is much smaller than elsewhere at the same latitude, resulting in a relatively dense and extensive area suitable for *Nicotiana tabacum* L., since its annual precipitation was in the range of the most suitable value of annual precipitation (bio12) obtained in this study (**Figure 4B**).

China, sharing the same northern temperate zone with Europe, however has a different situation. In China, the low mean temperature of ocean currents from the eastern Pacific plate keeps the average temperature of the coldest months in the eastern region below 0°C, while in the driest season, the climate in the eastern coastal area is typically above 35°C due to the return of ocean currents. The most suitable habitat for the growth of *Nicotiana tabacum* L. is therefore not concentrated in the southern coastal strip, but rather in the central and southwestern extension of the Yunnan-Guizhou Plateau (**Figure 5A**; **Supplementary Figure S3**). Yunnan has a tropical and subtropical monsoon climate with distinct three-dimensional climatic characteristics, which shows small annual temperature differences (generally only 10°C to 12°C), distinct dry and wet seasons, and vertical temperature changes with altitude. The average temperature in July, the hottest month, is between 16.0 and 28.0°C, and the annual precipitation is between 1500 and 1750 mm. According to the results of this paper, the climatic characteristics of the Yunnan-Guizhou Plateau region are similar to the growing environment of *Nicotiana tabacum* L., with rainy summers and favorable temperatures throughout the year, which are very suitable for the introduction of different varieties of tobacco. Additionally, it’s worth noting that some habitat loss occurred in this region under SSP3-7.0 and SSP5-8.5 climate scenarios (**Supplementary Figures S2C**, **D**), giving us an early warning to track climate change and adjust cultivation practices and areas in time.

Prediction in this study also showed much highly suitable habitats in the United States (**Figure 5A**). The west coast of North America has a typical temperate maritime climate, the humid climate and suitable temperatures make this region a highly suitable habitat for tobacco. The vast central and eastern region of the United States is dominated by a subtropical humid monsoon climate and a temperate continental climate. Since the western Cordillera blocks moisture from the Pacific Ocean, most precipitation of the central and eastern region comes from the Atlantic Ocean. Therefore, in the central and eastern region, a decreasing trend of the suitability for *Nicotiana tabacum* L. occurs from east to west (**Figure 5A**). Overall, the climate advantage, combined with the advanced large-scale farming, has made the United States the world’s leading producer of high-quality tobacco.

Finally, let’s think about the threat of global warming. It has resulted in regional and seasonal shifts in precipitation patterns, which are expected to increase in high latitude and monsoon regions due to greenhouse gas emissions (Cook et al., 2020). This is in agreement with the result of this study that the ecological distribution of *Nicotiana tabacum* L. in high latitudes in the interior of Eurasia will significantly expand along with the rising of the temperature from the SSP1-2.6 scenario, the SSP2-4.5 scenario, the SSP3-7.0 scenario to the SSP5-8.5 scenario in the future (**Figures 6A–D**).

*Nicotiana tabacum* L., as a cash crop, not only needs yield, but also needs the quality of the leaf. In actual production, the cultivation techniques, which was not considered in this study, are also important factors affecting growth. Therefore, based on the results of this study, a comprehensive evaluation of climate, soil, cultivation techniques, and even land policy is necessary before large-scale planting in practice.

## Data availability statement

The raw data supporting the conclusions of this article will be made available by the authors, without undue reservation.

## Author contributions

LJ: Methodology, Software, Validation, Writing – original draft. MS: Data curation, Investigation, Writing – review & editing. MH: Data curation, Writing – original draft. MY: Resources, Investigation, Writing – review & editing. MZ: Validation, Visualization, Writing – review & editing. HY: Funding acquisition, Resources, Supervision, Visualization, Writing – review & editing.
