# Supplementary material for: Study on the change of global ecological distribution of Nicotiana tabacum L. based on MaxEnt model
Source: Front Plant Sci. 2024 Jul 17;15:1371998. doi: 10.3389/fpls.2024.1371998 (PMC11292735; doi:10.3389/fpls.2024.1371998)
Supplement: Supplementary file 1 [file DataSheet_1.pdf]

Supplementary Material

Supplementary Tables

Table S1 The twenty-two environment variables used for the calculation of the MaxEnt model

Type of variable	Environment variables	Description
Bioclimatic	bio2	Mean Diurnal Temperature Range
	bio8	Mean Temperature of Wettest Quarter
	bio10	Mean Temperature of Warmest Quarter
	bio12	Annual Precipitation
	bio15	Precipitation Seasonality
	bio17	Precipitation of Driest Quarter
Topographic	DEM	Digital Elevation Model
	Slope	Slope Gradient
	Aspect	The Direction of the Slope Faces
Solar Radiation	Srad9	Solar Radiation in September
Soil	CEC_EFF	Cation Exchange Capacity Efficiency
	COARSE	Soil Sand Thickness
	CLAY	Clay Content
	ROOT_DEPTH	Root Soil Depth
	SILT	Silt Content
	ALUM_SAT	Aluminum Saturation
	CEC_CLAY	Cation Exchange Capacity of Clay
	CEC_SOIL	Cation Exchange Capacity of Soil
	ELEC_COND	Electrical Conductivity
	CN_RATIO	Carbon Nitrogen Ratio
	TCARBON	Calcium Carbonate Content
	SAND	Sand Content

Table S2 Contribution of environment variables to the construction of the MaxEnt model

Variables	Percent contribution/%	Permutation importance/%
bio10	28.9	34.3
bio12	28.8	16.4
Srad9	7.9	14.2
ELEC_COND	7.6	0.3
bio2	5.7	3.8
bio17	4.6	4.6
TCARBON	4.4	8.5
Slope	2.8	4.5
bio15	1.6	1.5
bio8	1.3	2.9
ROOT_DEPTH	1.2	0.4
ALUM_SAT	1.2	1.8
DEM	0.9	1.8
CEC_CLAY	0.7	0.7
SILT	0.6	0.9
SAND	0.5	0.8
CEC_EFF	0.5	0.8
CEC_SOIL	0.4	0.7
CLAY	0.2	0.9
COARSE	0.1	0.3
Aspect	0	0
CN_RATIO	0	0.1

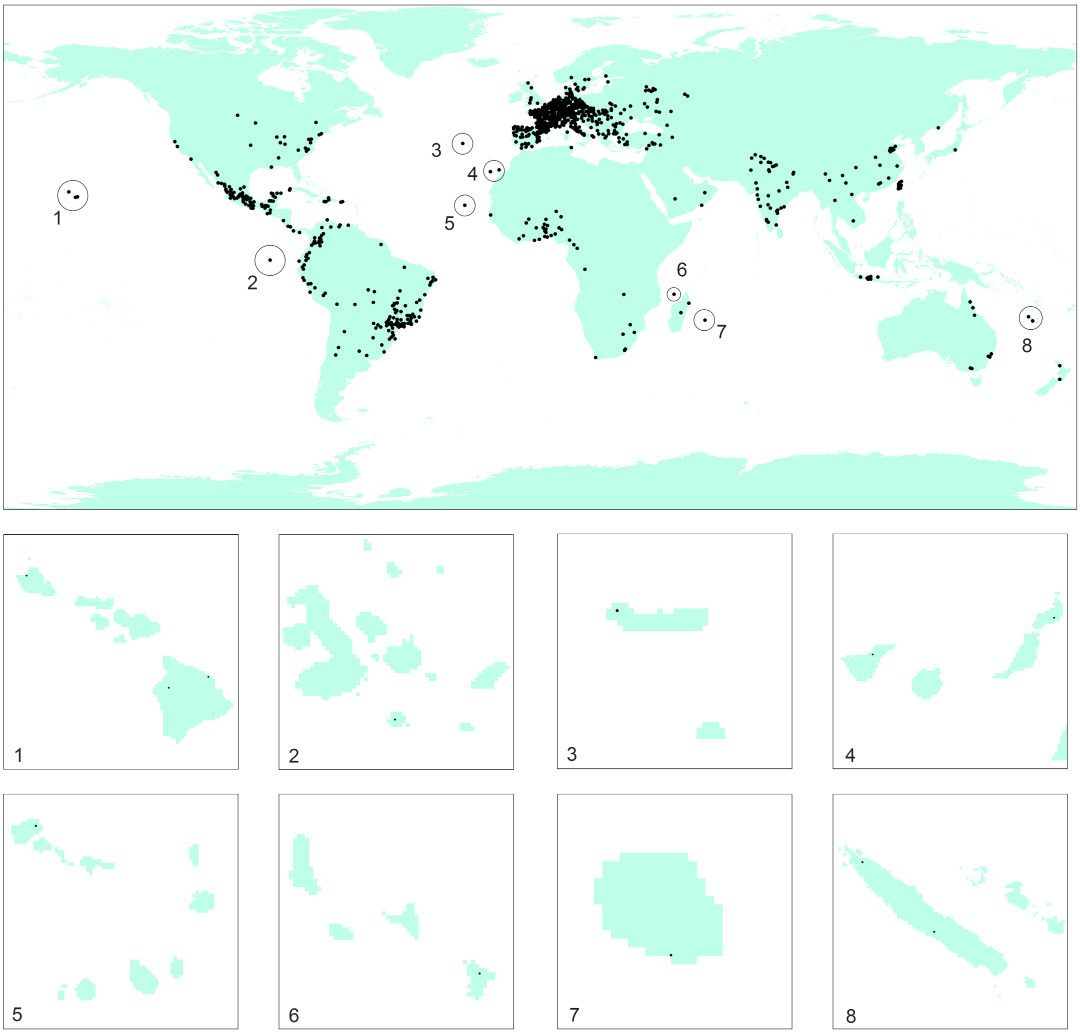

Figure S1. Sample records of the global distribution of *Nicotiana tabacum* L.. Black dots represent the 777 sample records of *Nicotiana tabacum* L. used for the final calculation of the MaxEnt model. The map was directly obtained from WorldClim (https://worldclim.org) and processed with ArcMap 10.8.

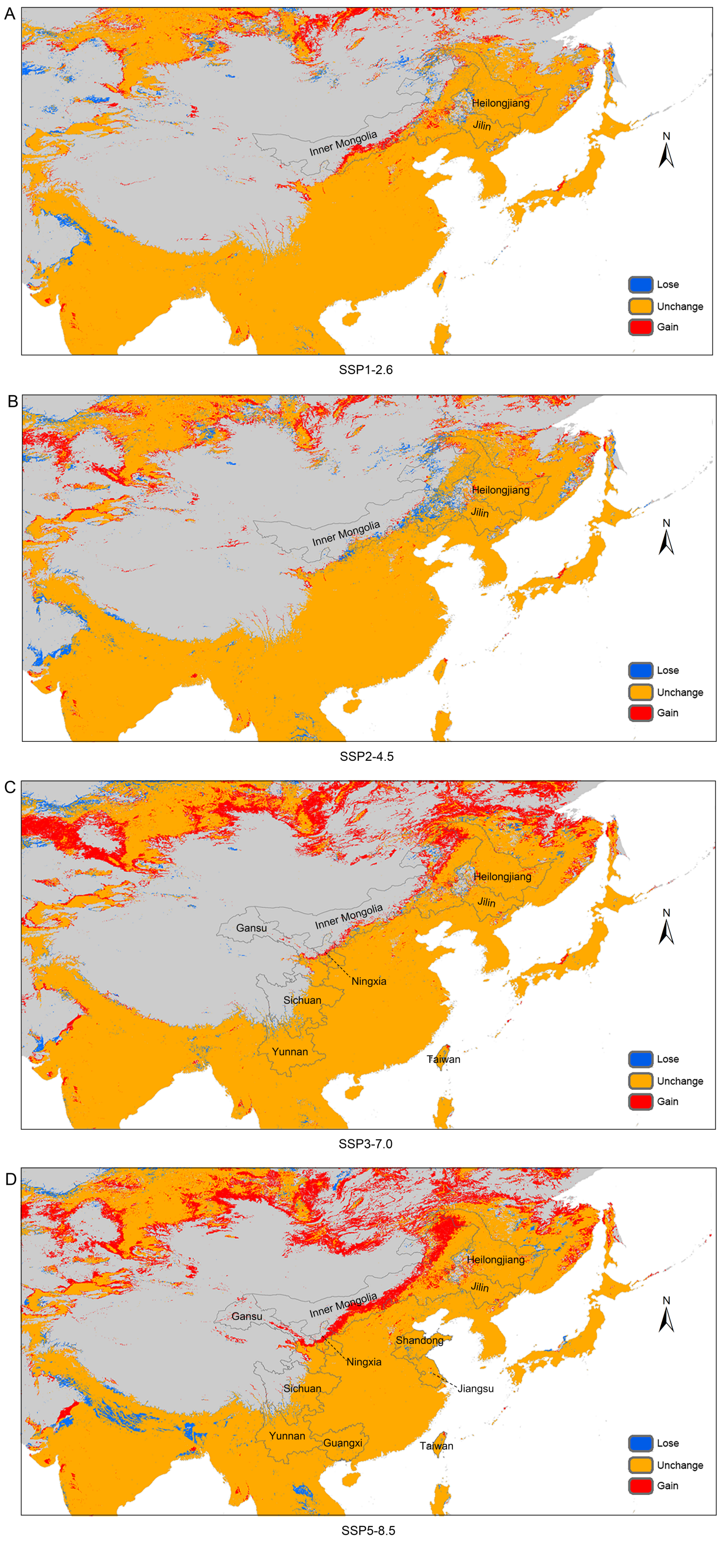

Figure S2 Change of ecological distribution of *Nicotiana tabacum* L. under four future climate scenarios in China. (A) Ecological distribution change under SSP1-2.6 climate scenario. (B) Ecological distribution change under SSP2-4.5 climate scenario. (C) Ecological distribution change under SSP3.7-0 climate scenario. (D) Ecological distribution change under SSP5-8.5 climate scenario.

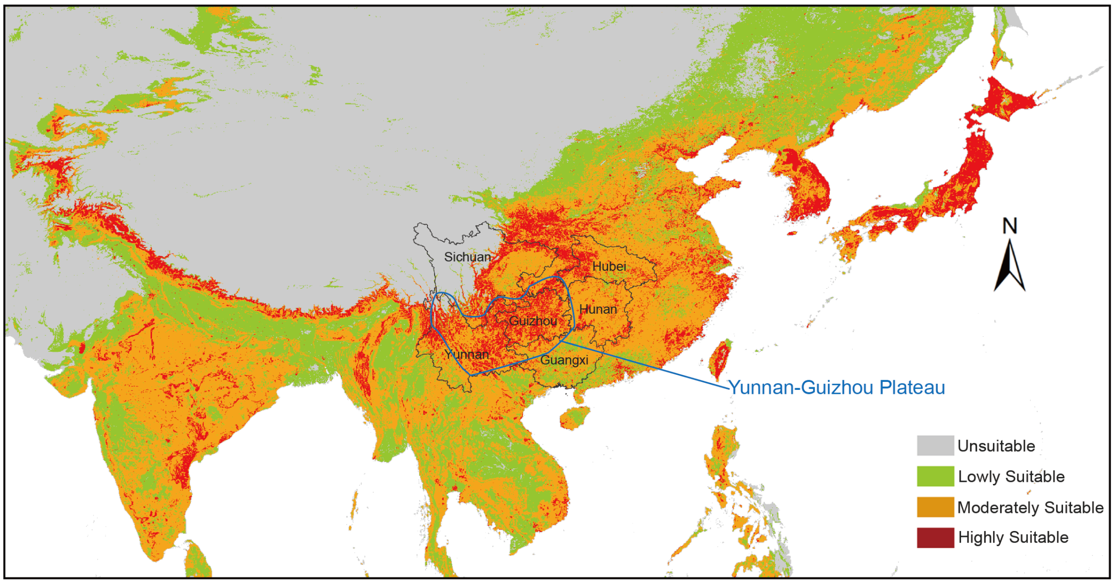

Figure S3 Suitable habitats of *Nicotiana tabacum* L. on the Yunnan-Guizhou Plateau, China, under current climate conditions.
